# miR-328-3p promotes migration and invasion by targeting H2AFX in head and neck squamous cell carcinoma

**DOI:** 10.7150/jca.60743

**Published:** 2021-09-09

**Authors:** Huiling Ma, Chao Liu, Shuiting Zhang, Wenhui Yuan, Junli Hu, Donghai Huang, Xin Zhang, Yong Liu, Yuanzheng Qiu

**Affiliations:** 1 Department of Otolaryngology Head and Neck Surgery, Xiangya Hospital, Central South University, Changsha, China.; 2 Otolaryngology Major Disease Research Key Laboratory of Hunan Province, Changsha, China.; 3 Clinical Research Center for Pharyngolaryngeal Diseases and Voice Disorders in Hunan Province, Changsha, China.; 4 National Clinical Research Center for Geriatric Disorders, Xiangya Hospital, Central South University, Changsha, China.

**Keywords:** Head and neck squamous cell carcinoma, miR-328-3p, Migration, Invasion, H2AFX

## Abstract

Migration and invasion are the initial step in the metastatic process, while metastasis is responsible for the poor prognosis of head and neck squamous cell carcinoma (HNSCC). Since miRNA has been found as an important regulator of gene expression at the post-transcriptional level in various diseases including carcinoma, exploring the role of miRNA in cancer metastasis will facilitate the target therapy of advanced HNSCC. MiR-328-3p has been reported to be an onco-miRNA or a tumor suppressor in several cancers. However, the role of miR-328-3p in HNSCC migration and invasion remains undefined. In this study, we first demonstrated that miR-328-3p enhanced migration and invasion of HNSCC *in vitro*, accompanying with a promotion of epithelial-mesenchymal transition (EMT) and mTOR activity. Meanwhile, we confirmed that miR-328-3p directly targeted the 3'UTR of H2A histone family, member X (H2AFX), which served as a tumor suppressor in migration and invasion of HNSCC. Moreover, H2AFX could partially reverse the migration and invasion of HNSCC caused by miR-328-3p. Overall, our results indicated that miR-328-3p enhanced migration and invasion of HNSCC through targeting H2AFX and activated the mTOR pathway.

## Introduction

Head and neck squamous cell carcinoma (HNSCC) accounted for more than 800,000 new cancers and 450,000 deaths in 2018 1, 2. Multidisciplinary treatment involving a combination of surgery, radiation, and chemotherapy are the effective therapies for most HNSCC patients. Unfortunately, once HNSCC has spread outside of the head and neck region, treatment is seldom curative. Thus, new targeted agents against migration and invasion can provide crucial countermeasures to improve the prognosis of patients with HNSCC.

MicroRNAs (miRNAs) serve as important regulators of gene expression at the post-transcriptional level through interacting with 3′-end and more rarely with 5′-end of mRNA transcribed from target genes 3. There are over 60% human protein-coding genes paired with miRNAs^4^. Numerous studies over the past decade have been devoted to assess the role of miRNAs in various diseases, and have found that miRNAs serve as crucial onco-miRNAs or tumor suppressor miRNAs in cancer 5-9. Thus, the role of miRNAs in cancer could not be ignored.

MiR-328-3p has been reported to be dysregulated in various diseases, such as pulmonary arterial hypertension, multiple sclerosis and epilepsy, as well as cancers 10-12. Recent studies showed that miR-328-3p may prevent tumor progression in non-small cell lung cancer, colorectal cancer, hepatocellular carcinoma, bladder cancer and osteosarcoma 13-17, but served as an onco-miRNA in ovarian cancer 18, which demonstrated the complicated roles of miR-328-3p in different types of tumors. In regards to HNSCC, the effect of miR-328-3p on migration and invasion of HNSCC remains unclear. In this study, we showed that miR-328-3p could promote migration and invasion by targeting H2AFX in HNSCC and activate the mTOR pathway, which provide novel insights for elucidating the molecular mechanisms involving migration and invasion of HNSCC.

## Materials and methods

### Cell culture

The Tca8113, a cell line from human tongue squamous carcinoma, was bought from the Cell Bank of Type Culture Collection of the Chinese Academy of Sciences. Tu686 cell line, established from a primary tongue tumor, was kindly provided by Dr. Zhuo Chen (Emory University Winship Cancer Institute, Atlanta, Georgia, USA). FaDu, the Human hypopharyngeal squamous cell carcinoma cell line, was purchased from American Type Culture Collection (ATCC, VA, USA). JHU011, the human laryngeal squamous cell carcinoma cell line, was obtained from Division of Head and Neck Cancer Research at the Johns Hopkins University. Dulbecco's modified Eagle's medium (DMEM; Gibco, USA) was the culture substrate of FaDu, while RPMI Medium 1640 basic (Gibco, USA) was used for Tca8113 and JHU011. Tu686 cells were cultured in DMEM/F12 medium (Gibco, USA). All these media were supplemented with 10% fetal bovine serum (FBS; Gibco, USA) and 1% Penicillin-Streptomycin. Cells were incubated at 37 °C in 5% CO_2_.

### Transfection

Tca8113 and FaDu cells were transfected with miR-328-3p mimics (Genepharma, Suzhou, China) or negative control (NC), while JHU011 and Tu686 cells were transfected with miR-328-3p anti-sense molecules (also called inhibitor) or NC, using the si-Mate^TM^ (Genepharma) according to the manufacturer's protocol. Tca8113 and FaDu cells were transfected with H2AFX cDNA plasmid (EX-B0074-M98, GeneCopoeia Inc., MD, USA) using the FuGENE® 6 (Promega, Madison, WI, USA).

### Transwell invasion assay

2×10^4^ cells were starved in 200 μl serum-free medium, then seeded in the upper transwell chamber (Corning) which was covered with 200 μg/mL of Matrigel (Corning, NY), and 600 μl complete medium containing 10% FBS was added in the lower chamber at the same time. After 48 hours' incubation, cells invaded on the lower side of top chamber were fixed with 4% paraformaldehyde (Biosharp) for 20 min and stained with crystal violet (Beyotime) for 15 min. Cells on upper side of top chamber were softly removed by a cotton swab. Take pictures randomly under 50×, 100×, 200× Leica fluorescence microscope (Wetzlar, Germany). The invading cells were counted by Image J 19.

### Wound healing assay

Wound healing assay was used to evaluate the ability of cell migration. Cells were seeded on a six-well plate with over 90% confluency. Then using a sterile micropipette tip to create scratch wound with the same width in each group. Cells were cultured in serum-free medium for at least 24 hours. Images were captured under an inverted microscope, and the wound closure areas were calculated by Image J 19.

### Cell proliferation assay

Cell proliferation based on cell viability was assessed by using a Cell Counting Kit (Meilunbio). 3×10^3^ cells were seeded on 96-well plates after transfection. After 0, 24, 48, 72, 96 hours, cells were co-incubated with 10 μl CCK8 and 100 μl serum-free medium per well at 37 °C in 5% CO_2_ incubator for 1 hour, while the medium's absorbance without a cell as blank control. The absorbance at 450 nm was detected using a Model 680 Microplate Reader (BioTek).

### Colony formation assay

Colony formation assay was used to determine the multiplication capacity of HNSCC cells. 400 cells were seeded in each well of six-well plates. About 2 weeks later, cells were fixed with 4% paraformaldehyde (Biosharp) for 20 min and stained with crystal violet (Beyotime) for 15 min. Colonies with over 50 cells were counted by Image J 20.

### Data source and EMT score

Publicly available dataset of HNSCC cohort, including gene expression profile of 522 HNSCC patients, was downloaded from TCGA data portal (http://cancergenome.nih.gov/). Based on the expression level of 76-gene EMT signature obtained from the study of *Lauren Averett Byers et al.* 21, every HNSCC sample got an EMT score, which was calculated as the average expression level of “mesenchymal” genes minus the average expression level of “epithelial” genes. The 522 tumor samples were ranked by EMT score from lowest to highest, then the first 174 samples were defined as “epithelial” HNSCC while the last 174 samples were defined as “mesenchymal” HNSCC.

### Quantitative reverse transcription polymerase chain reaction (qRT-PCR)

Total RNA was extracted from cell lines using the AG RNAex Pro reagent (Accurate Biology). 2 μg total RNA was used to synthesized single-stranded cDNA by All-in-OneTM miRNA or mRNA cDNA synthesis kit (GeneCopoeia Inc., MD, USA). qRT-PCR was carried out on the ABI QuantStudio 7 Flex Real-Time PCR System using All-in-One Kit (GeneCopoeia Inc., MD, USA) and sets of gene-specific primers. The reaction parameters included an initial step at 95 °C for 10 min, 40 cycles of 95 °C for 10 seconds, 62.5 °C for 20 seconds, and 72 °C for 15 seconds. U6 and GAPDH were used as endogenous control for miRNA and mRNA, respectively, and data were gauged through the classical 2^-ΔΔCt^ method. Sequences of primers used in this study were listed in Table S1.

### Western blotting

Total protein was extracted by using RIPA lysis buffer (NCM Biotech) with protease inhibitor cocktail and phosphatase inhibitor after transfection for 72 hours. 16-30 μg protein was loaded and electrophoresed on 8-12% SDS-PAGE gels, then transferred to a PVDF membranes (Millipore, Bedford, MA, USA). After sealing in NcmBlot blocking buffer (NCM Biotech) for 15 min, the membrane was incubated within primary antibody overnight at 4 °C. Then a horseradish peroxidase-conjugated secondary antibody (1:2000, Cell Signaling Technology) was used. Target protein bands were visualized using super sensitive ECL luminescence reagent (Dalian Meilun Biotechnology Co, Ltd.) in a Chemiluminescence and Fluorescence Imaging System (Bio-Rad). The antibodies used in this study were listed in Table S2.

### miRNA target prediction

The potential targets of miR-328-3p were predicted with the Encyclopedia of RNA Interactomes (ENCORI, http://starbase.sysu.edu.cn/) database, which include six algorithms (PITA, miRmap, microT, miRanda, Pictar and TargetScan). And ENCORI was also used to acquire the predicted binding sites between miR-328-3p and the target gene.

### Dual-luciferase activity assay

Cells were seeded into 24-well plate, and co-transfected with 50 nM of either miR-328-3p mimics or NC oligos in combination with 150 ng of wild-type or mutated 3'-UTR H2AFX plasmids (GeneCopoeia Inc., MD, USA) via si-MateTM (Genepharma) and the FuGENE® 6 (Promega, Madison, WI, USA), respectively. Then, the luciferase activity was appraised through utilizing the dual-luciferase assay system (Promega Corporation, WI) on the basis of the manufacturer's information.

### Statistical analyses

GraphPad Prism (version 5.01, GraphPad Software, Version X; La Jolla, CA) was used for statistical analysis of the data. Unpaired t-test and Mann-Whitney U test were performed to analyze the significance of differences between groups. P-values less than 0.05 were considered as statistically significant.

## Results

### Overexpression of miR-328-3p promotes HNSCC migration and invasion

**Figure 1:**
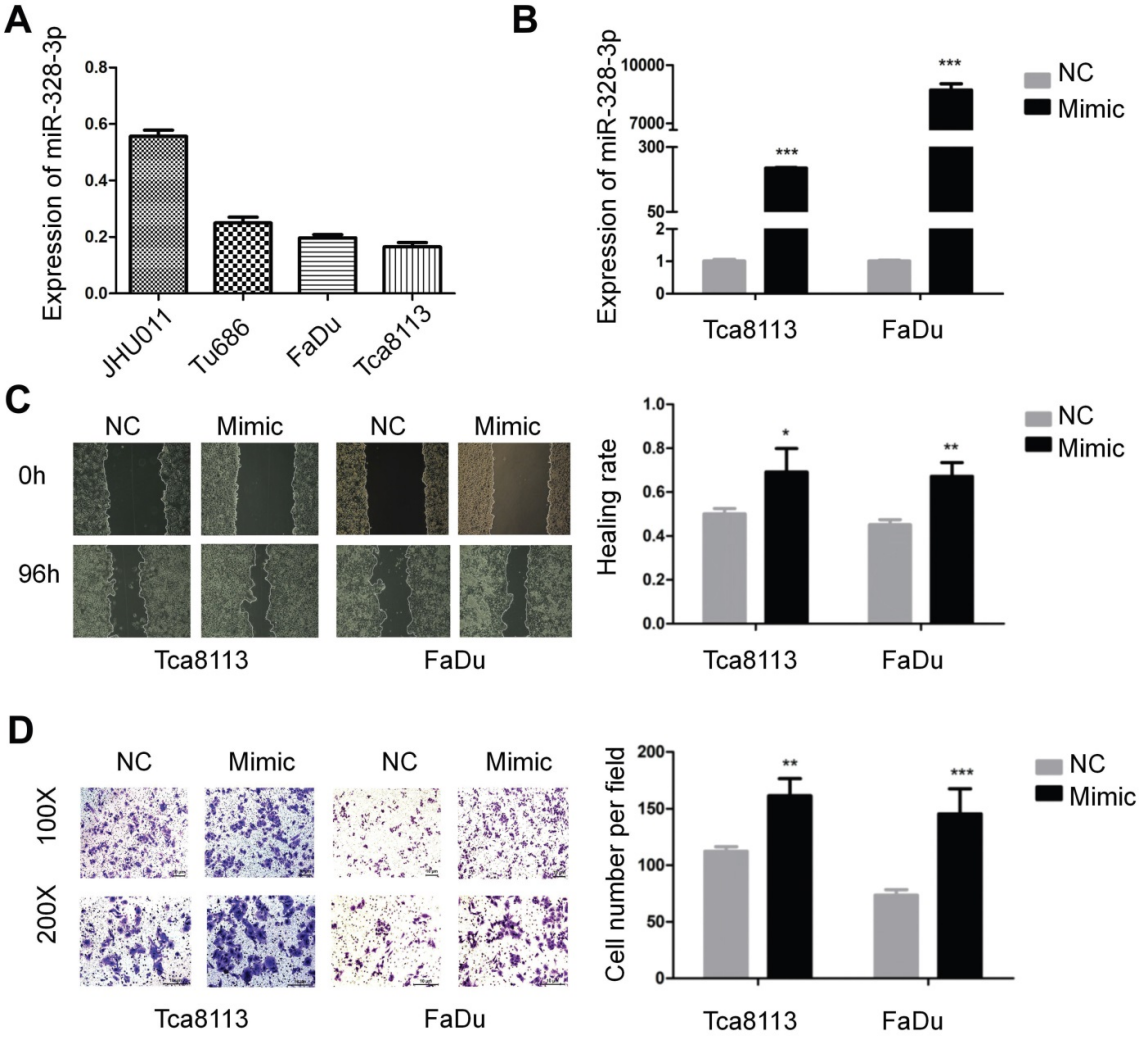
Overexpression of miR-328-3p promoted migration and invasion in HNSCC. **A.** Different expression levels of miR-328-3p in HNSCC cell lines. **B.** miR-328-3p expression was significant upregulated in Tca8113 and FaDu cells after transfection with miR-328-3p mimic. **C** and** D**. Wound healing assays (**C**) and transwell invasion assays (**D**) showed overexpression of miR-328-3p could promote the ability of migration and invasion in Tca8113 and FaDu cells. NC: negative control. Each experiment was repeated in triplicate. Data are presented as the mean ± SD. Student's unpaired t-test, *, P <0.05; **, P < 0.01; ***, P < 0.001.

Firstly, we investigated the basic expression level of miR-328-3p in four HNSCC cell lines and found that the expression of miR-328-3p was higher in JHU011 and Tu686, and lower in Tca8113 and FaDu (Fig. 1A), which were selected for loss and gain of function experiments, respectively.

For the gain of function experiments, miR-328-3p mimic was transfected in the Tca8113 and FaDu cells and a significant overexpression of miR-328-3p was confirmed by qRT-PCR (Fig. 1B). The wound healing and transwell invasion assays showed that overexpression of miR-328-3p significantly increased the ability of migration and invasion in HNSCC cells (Fig. 1C, D). Furthermore, we found that the elevated miR-328-3p had no obvious effect on the proliferation of HNSCC cells via cell proliferation assay and colony formation assay (Fig. S1A, B).

### Knockdown of miR-328-3p suppresses HNSCC migration and invasion

**Figure 2:**
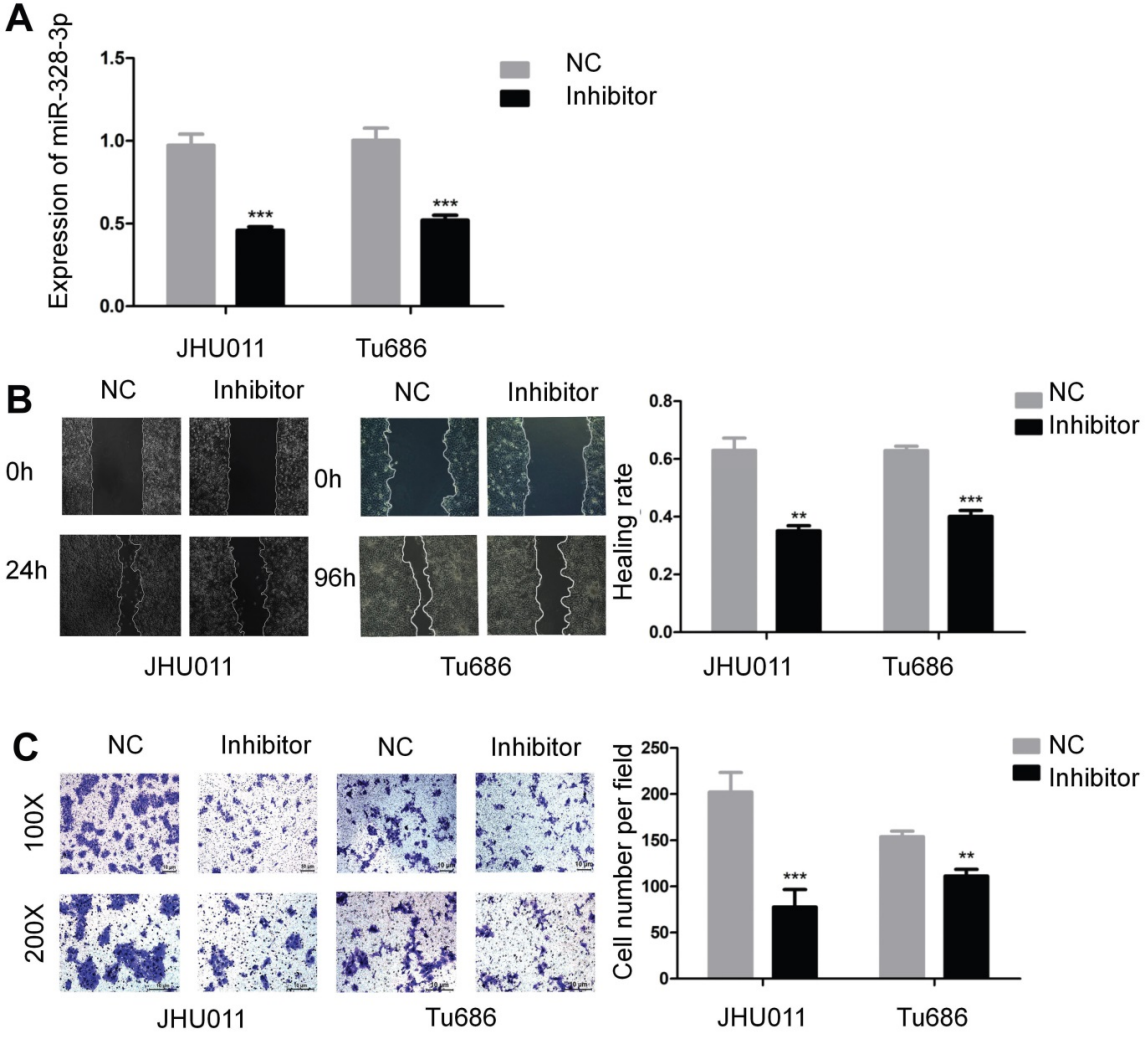
Downregulation of miR-328-3p suppressed migration and invasion in HNSCC. **A.** miR-328-3p expression was significantly inhibited in JHU011 and Tu686 cells after transfection with miR-328-3p anti-sense molecules (inhibitor). **B** and **C**. Wound healing assays (**B**) and transwell invasion assays (**C**) showed down-regulation of miR-328-3p could suppress the ability of migration and invasion in JHU011 and Tu686 cells. Each experiment was repeated in triplicate. All data are represented as the mean ± SD. Statistical analysis was performed using Student's t- test. *, P <0.05; **, P < 0.01, ***, P < 0.001.

To further explore the function of miR-328-3p in HNSCC, miR-328-3p anti-sense molecules (inhibitor) was adopted to silence the expression of miR-328-3p. qRT-PCR showed that following transfection of inhibitor for 48 hours, the expression level of miR-328-3p was down-regulated by at least 50% in JHU011 and Tu686 cells (Fig. 2A). Then the wound healing and transwell invasion assays demonstrated that silencing miR-328-3p suppressed cell migration and invasion of JHU011 and Tu686 cells (Fig. 2B, C).

### Upregulation of miR-328-3p promotes EMT in HNSCC

**Figure 3:**
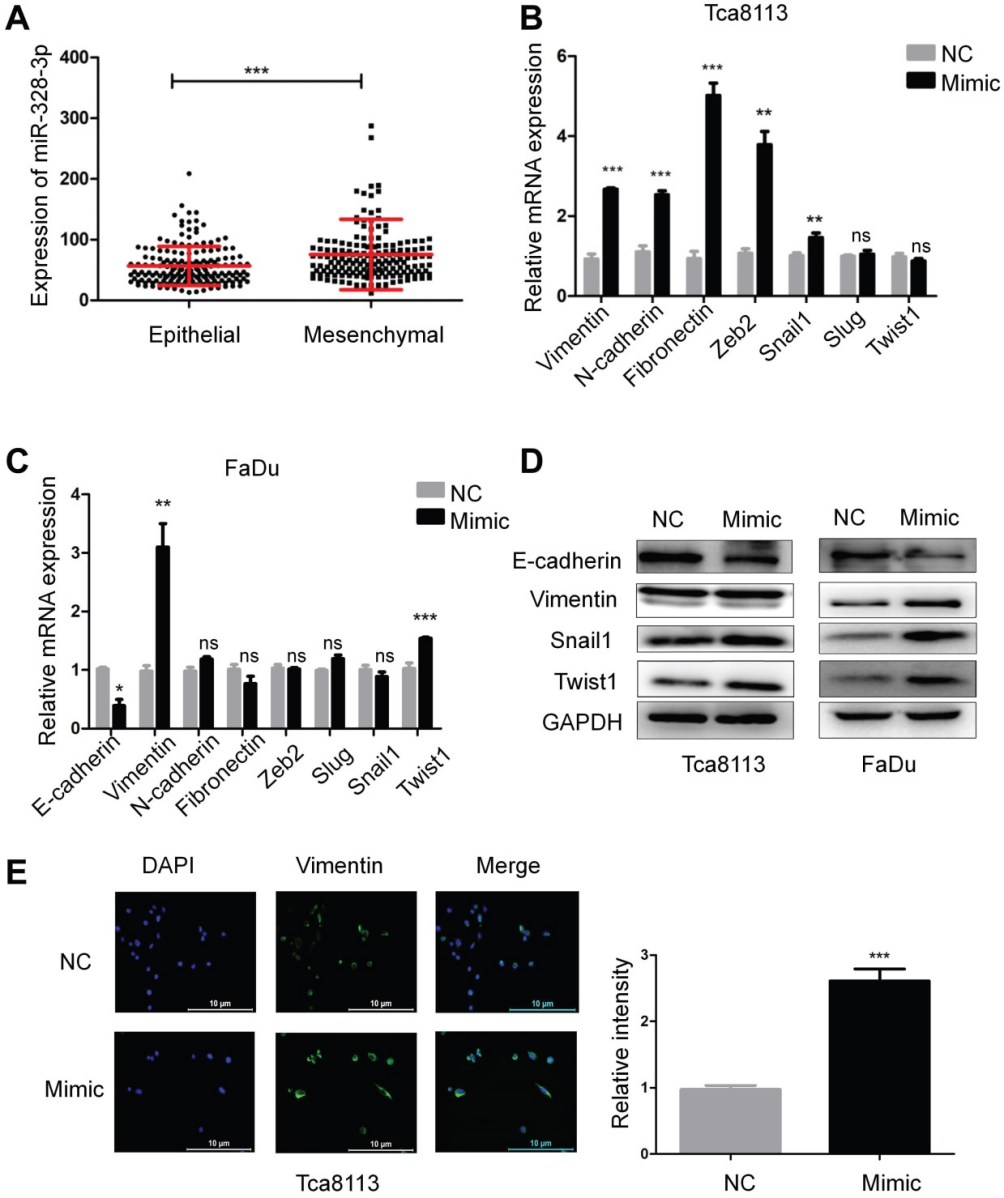
Overexpression of miR-328-3p promoted EMT in HNSCC. **A.** The expression of miR-328-3p in “Epithelial” or “Mesenchymal” HNSCC in TCGA dataset. **B-D.** qRT-PCR (**B and C**) and Western blotting (**D**) were used to quantify the expression of the EMT markers of E-cadherin, Vimentin, Fibronectin and the related transcription factors Snail1, Slug, Zeb2 and Twist1 in Tca8113 and FaDu cells. GAPDH was used as a loading control. **E.** Representative immunofluorescence staining images showed the expression level of Vimentin in Tca8113 cells. ns, not significant. Each experiment was repeated in triplicate and all data are presented as mean ± SD. Mann-Whitney U test was used for Figure 3A, while Student's unpaired t-test was used for the others. *, P < 0.05; **, P < 0.01; ***, P < 0.001.

Epithelial-mesenchymal transition (EMT) plays a key role during carcinoma pathogenesis by imparting the mesenchymal phenotypes associated with the cells of highly aggressive tumors 22. According to the scoring method of tumor EMT status 23, the expression of miR-328-3p in “Mesenchymal” HNSCC was higher than that in “Epithelial” HNSCC (Fig. 3A). To further explore the correlation between miR-328-3p and EMT in HNSCC, we detected the mRNA and protein level of hallmarks of EMT, such as epithelial cadherin (E-cadherin), Vimentin, neural cadherin (N-cadherin) and transcription factors that repress the epithelial phenotype and activate the mesenchymal phenotype. The results of qRT-PCR showed that miR-328-3p overexpression in Tca8113 cells resulted in the upregulation of mesenchymal marker Vimentin, N-cadherin and Fibronectin, with the increase of transcription factors Snail1, Zeb2 (Fig. 3B). Meanwhile, in FaDu cells, upregulated miR-328-3p decreased the expression of E-cadherin and promoted the expression of Vimentin and Twist1 (Fig. 3C). Additionally, Western blotting was used to confirm that miR-328-3p overexpression suppressed the levels of E-cadherin and elevated the levels of Vimentin, Snail1 and Twist1 in both Tca8113 and FaDu cells (Fig. 3D, Fig. S2A-B). In addition, immunofluorescent analysis confirmed that miR-328-3p overexpression elevated the levels of Vimentin in Tca8113 cells (Fig. 3E). These results indicated that upregulation of miR-328-3p promoted EMT in HNSCC.

### Overexpression of miR-328-3p activates the mTOR pathway

**Figure 4:**
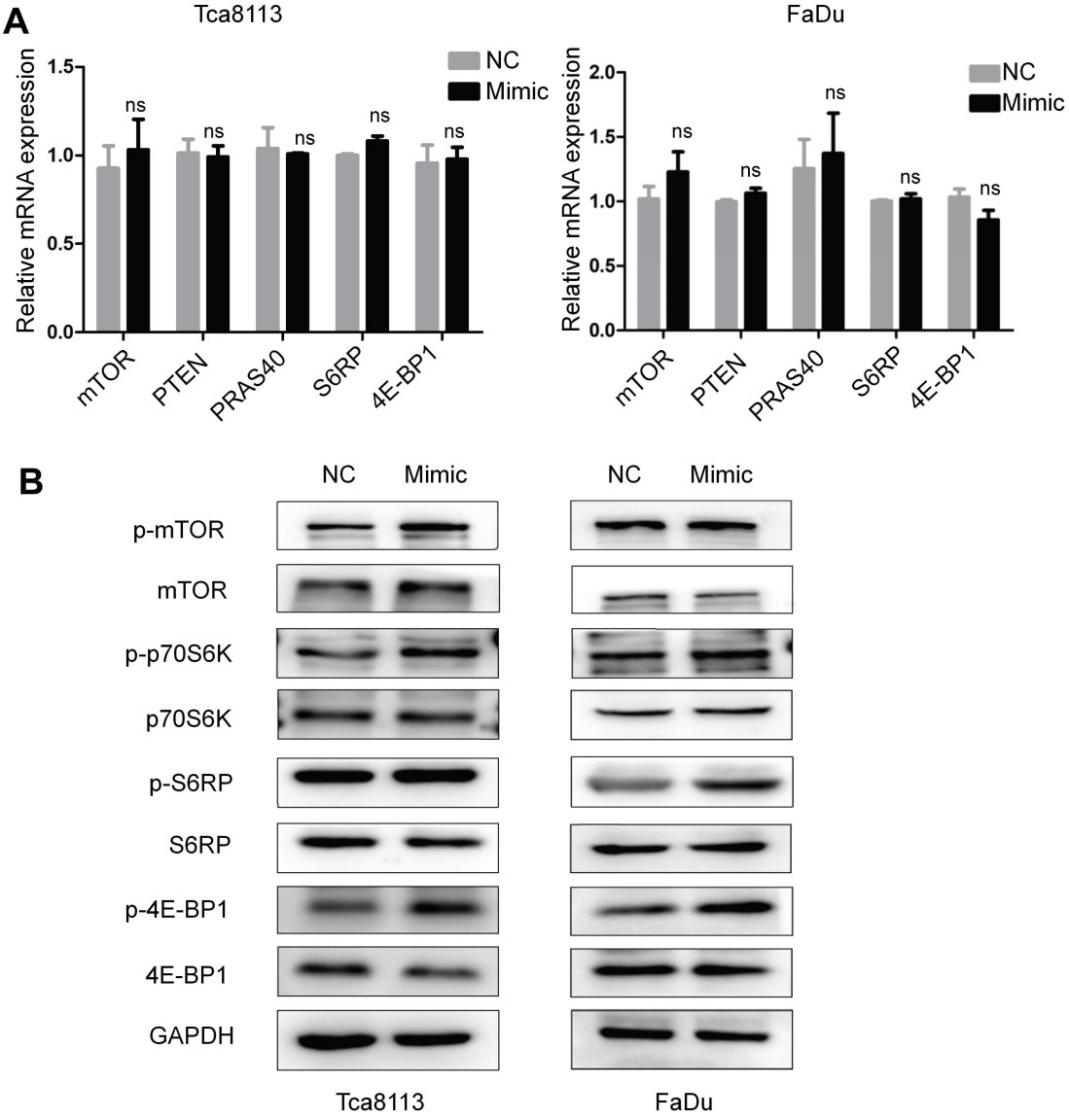
Overexpression of miR-328-3p activated the mTOR pathway in HNSCC. **A and B**. Following miR-328-3p overexpression in Tca8113 and FaDu cells, qRT-PCR (**A**) showed the mRNA expression of the markers of mTOR pathway, including mTOR, PTEN, PRAS40, S6RP and 4E-BP1 did not change significantly. Western blotting (**B**) showed the phosphorylated proteins level of mTOR, p70S6K, S6RP and 4E-BP1 were elevated. GAPDH served as an internal control.

Previous studies have demonstrated that the mTOR pathway may be associated with EMT of carcinomas 24. To further investigate whether miR-328-3p could exert its effect through mTOR signaling pathway, the mRNAs level and the phosphorylated protein level of mTOR, p70S6K, S6RP and 4E-BP1 were determined by qRT-PCR (Fig. 4A) and Western blotting (Fig. 4B) in Tca8113 and FaDu cells transfected with miR-328-3p mimic. The phosphorylated protein level was upregulated in miR-328-3p-mimic group while the non-phosphorylated proteins showed no obvious differences, suggesting that miR-328-3p could activate the mTOR pathway in HNSCC.

### H2AFX is a direct target of miR-328-3p

**Figure 5:**
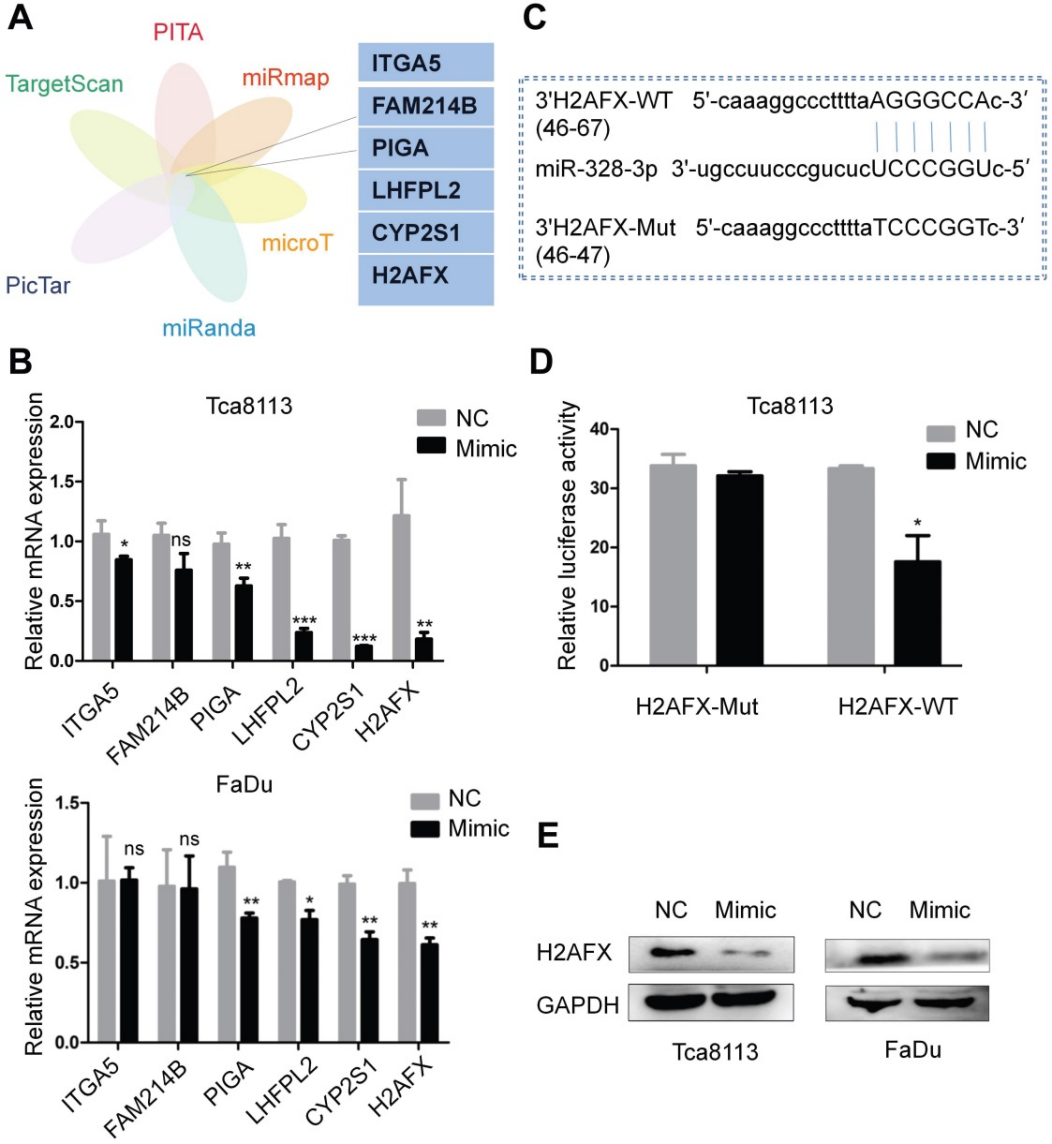
H2AFX was a direct target of miR-328-3p. **A.** Potential target genes were obtained by integrating six algorithms (PITA, miRmap, microT, miRanda, Pictar and TargetScan) in ENCORI. **B.** The relative mRNA expression of potential target genes after transfecting with miR-328-3p mimic was examined by qRT-PCR in Tca8113 and FaDu cells. **C.** Predicted binding site between miR-328-3p and the wild-type 3'-UTR of H2AFX was obtained by ENCORI. **D.** Dual luciferase reporter assay conducted in Tca8113 cells confirmed that H2AFX was a direct target of miR-328-3p. **E.** Western blotting showed that H2AFX protein expression was down-regulated in Tca8113 and FaDu cells after transfecting with the miR-328-3p mimic. Each experiment was repeated in triplicate. Data are presented as the mean ± SD. Student's unpaired t-test. *, P < 0.05; **, P < 0.01; ***, P < 0.001.

To explore the target genes of miR-328-3p, we selected candidate genes at ENCORI website, which included data from six algorithms (PITA, miRmap, microT, miRanda, Pictar and TargetScan) (Fig. 5A). H2AFX was selected as the target gene owing to its most obvious downregulation of mRNA expression compared with the other candidate genes after transfecting with miR-328-3p mimic in Tca8113 and FaDu cells (Fig. 5B). Then the luciferase reporter assay further confirmed the direct binding of miR-328-3p to the 3'UTR of H2AFX mRNA (Fig. 5C, D). Western blotting validated the dysregulation of H2AFX protein by miR-328-3p in Tca8113 and FaDu cells (Fig. 5E). These data showed that H2AFX is a direct target of miR-328-3p in HNSCC.

### H2AFX is partially involved in the migration and invasion of HNSCC mediated by miR-328-3p

**Figure 6:**
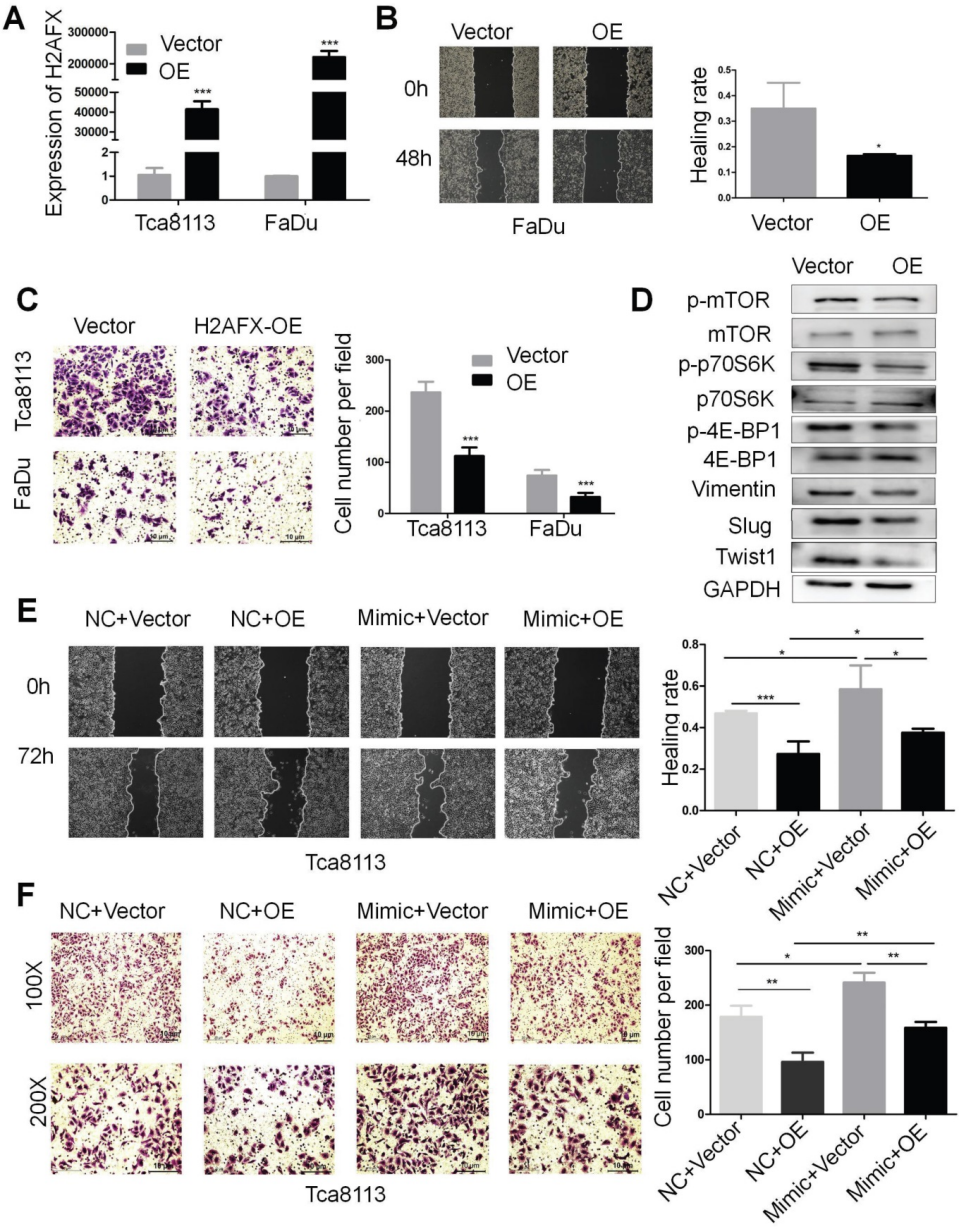
H2AFX was partially involved in the migration and invasion of HNSCC mediated by miR-328-3p. **A.** H2AFX mRNA expression was significant upregulated in Tca8113 and FaDu cells after transfection with H2AFX plasmid.** B and C.** Following H2AFX overexpression in Tca8113 and FaDu cells, wound healing (**B**) and transwell invasion assays (**C**) were performed. **D.** Western blotting showed the alternation of marker proteins for EMT and mTOR pathway. **E and F.** Cells transfected with miR-328-3p mimics or NC were subsequently treated with H2AFX plasmid or vector. Wound healing assays (**E**) and transwell invasion assays (**F**) showed overexpression of H2AFX could rescue the inhibitory effect on cell migration and invasion caused by the miR-328-3p-mimic in Tca8113 cells. OE, H2AFX overexpression. Each experiment was repeated in triplicate. Data are presented as the mean ± SD. P-values were calculated using the Student's t-test. *, P <0.05; **, P < 0.01.

In order to investigate the role of H2AFX in HNSCC, the H2AFX cDNA plasmid was transfected in Tca8113 and FaDu cells and the expression of H2AFX mRNA was successfully upregulated (Fig. 6A). Then, the wound healing and transwell invasion assays showed that upregulation of H2AFX suppressed the migration and invasion of HNSCC (Fig. 6B, C). Additionally, H2AFX overexpression significantly reduced the protein levels of Vimentin, Slug and Twist1 in Tca8113 cells, and the biomarkers of mTOR pathway including p-mTOR, p-p70S6K and p-4E-BP1 were also decreased (Fig. 6D, Fig. S2C).

To finally ascertain whether upregulation of H2AFX is responsible for the inhibition of miR-328-3p-mediated migration and invasion of HNSCC, Tca8113 cells were treated with H2AFX overexpressed plasmid and empty vector following the transfection with miR-328-3p-mimic and NC. As expected, the migration and invasion capacities of HNSCC cells co-transfected with miR-328-3p mimic and H2AFX plasmid was weaker than cells co-transfected with miR-328-3p mimic and empty vector, suggesting that H2AFX partially reversed the promotion effect on cell migration and invasion caused by the miR-328-3p in Tca8113 cells. In addition, HNSCC cells co-transfected with miR-328-3p mimic and H2AFX plasmid exhibited stronger migration and invasion capacities than those co-transfected with NC and H2AFX plasmid, which again proved that miR-328-3p can promote the migration and invasion of HNSCC cells (Fig. 6E, F). Moreover, H2AFX partially reversed the protein change of EMT biomarkers caused by miR-328-3p (Fig. S3A). These findings demonstrated that H2AFX was partially involved in miR-328-3p mediated migration and invasion in HNSCC.

## Discussion

MicroRNA plays a crucial role in cancer migration and invasion by incorporating within an RNA-induced silencing complex (RISC) and inducing post-transcriptional gene silencing by base pairing with a target mRNA and interfering with its translation to protein 6, 25. Several studies have revealed the function and mechanism of miR-328-3p on cancer migration and invasion. For examples, in osteosarcoma, colorectal cancer, liver cancer and breast cancer, miR-328-3p overexpression inhibited their migration, invasion, and EMT by directly inhibiting MMP-16, Girdin, Endoplasmic Reticulum Metallo Protease 1 (ERMP1) and CD44, respectively 17, 26-29. Intriguingly, there was an opposing effect of miR-328-3p in ovarian cancer, in which miR-328-3p overexpression could maintain the CSC population and promote tumor metastasis 18. Then a recent study revealed that miR-328-3p may promote bone metastasis by activating Wnt/β-catenin pathway in non-small cell lung cancer 30. Another study showed that hypoxic bone marrow mesenchymal cell (BMSC)-derived extracellular vesicles could deliver miR-328-3p to lung cancer cells to target the NF2 gene, which ultimately promoted the invasion, migration and EMT of lung cancer by inhibiting the Hippo pathway 31. These studies indicated that the function of miR-328-3p could be tissue- and tumor-specific, which was further confirmed by our study. In the current study, miR-328-3p served as an onco-miRNA and promoted the migration and invasion of HNSCC.

H2AFX is a variant of the H2A protein family, a component of the histone octamer in nucleosomes 32. H2AFX would be phosphorylated on residue serine 139 in cells triggered by double-stranded breaks (DSB) 33. γ-H2AFX can recruit DNA repair proteins and other DNA double strand breaks signaling to the damaged site. Once DNA is repaired, γ-H2AFX is dephosphorylated, thereby γ-H2AFX is widely used as a DNA damage marker *in vitro* 34, 35. For the role of H2AFX in cancer, researchers found that H2AFX can help prevent aberrant repair of both programmed and general DNA breakage and serves as a suppressor of genomic instability and tumors in mice, which is dosage-dependent 36. Moreover, loss of a single H2AFX allele compromises genomic integrity and enhances the susceptibility to cancer in the absence of p53 37, and H2AFX variants are associated with an increased risk of breast cancer 38, 39. Based on these results, H2AFX may serve as a tumor suppressor. However, in lung adenocarcinoma, patients with low expression of H2AFX have a significantly better overall survival 40, and H2AFX can promote radioresistance in non-small cell lung cancer and osteosarcoma 13, 41, which reveal that H2AFX may also be a tumor promoter. Despite these previous findings, studies involved the role of H2AFX in cancer migration and invasion and EMT remained scarce, and our study was the first to demonstrate that H2AFX functioned as a tumor suppressor to inhibit HNSCC migration and invasion *in vitro*. Additionally, upregulation of H2AFX partially abrogated increased migration and invasion induced by miR-328-3p, suggesting that H2AFX was a major target of miR-328-3p in HNSCC.

The mTOR signaling pathway encompasses two functionally distinct protein complexes: mTOR complex 1 (mTORC1) and mTOR complex 2 (mTORC2) 42, and only the former one is sensitive to the macrolide fungicide rapamycin. The mTORC1 contains two positive regulatory subunits, raptor and mLST8, and two negative regulators, PRAS40 and DEPTOR 43. The p70 ribosomal S6 kinase/S6 ribosomal protein (P70S6K/S6RP) and eukaryotic translation initiation factor 4E-binding protein 1 (4E-BP1) are two downstream targets of mTORC1 44. The mobilized P70S6K will induce the S6 activation, which is essential for protein synthesis 45, 46. In quiescent cells, 4E-BP1 competes with eIF-4G for binding to eIF-4E and represses translation by displacing the initiation factor 4F from the mRNA. Once 4E-BP1 is phosphorylated, its affinity for eIF-4E will decrease and release the block on cap-dependent translation 47. The mTORC2 activates the mTORC1 by activating Akt 48. Previous studies had showed that mTOR was not only a central regulator of cell growth, proliferation, differentiation and survival, but also played a critical role in the regulation of tumor cell motility, invasion and metastasis 49, 50. *Berven et al.* reported that Fibronectin, a mesenchymal marker, stimulated stress fiber formation in the absence of growth factors and caused an inactivation of p70S6K to regulate cell migration 51. *Lamouille et al.* found that the mTORC2 pathway was an essential downstream branch of TGF-β signaling. TGF-β can rapidly induce mTORC2 kinase activity in cells undergoing EMT, and control epithelial cell progression through EMT 52. *Liu et al.* found low expression of the apelin receptor promoted EMT in nasopharyngeal carcinoma cells by activating the PI3K-mTOR signaling pathway 53. These studies revealed that mTOR pathway was associated with EMT and tumor metastasis 54, 55. Additionally, several groups demonstrated anti-cancer effect of mTOR inhibitors in HNSCC xenografts either as a single agent or when combined with chemotherapy/radiotherapy 56-58. Clinical trials were also conducted to investigate the function of several mTOR inhibitors used either alone or in combination with chemotherapy or radiotherapy in HNSCC 59, 60. These results indicated that it was significant to explore the mechanism of mTOR pathway in HNSCC. In the current study, miR-328-3p can increase the phosphorylation protein level of mTOR, P70S6K, S6RP and 4E-BP1, and overexpression of the target gene H2AFX reduced the protein levels of p-mTOR, p-p70S6K and p-4E-BP1, suggesting that miR-328-3p/H2AFX axis could activate the mTOR pathway in HNSCC.

Taken together, our results demonstrated that miR-328-3p enhanced migration and invasion of HNSCC through targeting H2AFX and activated the mTOR pathway, which may be a potential therapeutic target in the treatment of HNSCC patients.

## Supplementary Material

Supplementary figures and tables.
