# Supplementary material for: miR-328-3p promotes migration and invasion by targeting H2AFX in head and neck squamous cell carcinoma
Source: J Cancer. 2021 Sep 9;12(21):6519–30. doi: 10.7150/jca.60743 (PMC8489127; doi:10.7150/jca.60743)
Supplement: Supplementary file 1 — Supplementary figures and tables. [file jcav12p6519s1.pdf]

Supplementary materials

Supplementary Figure S1. Overexpression of miR-328-3p did not affect the proliferation in HNSCC. **A and B.** Cell proliferation was unaffected following the upregulation of miR-328-3p through cell proliferation assay (**A**) and colony formation assays (**B**).

Supplementary Figure S2. Relative protein expression**. A** and **B.** The densitometric analyses of figure 3D by Image J. **C.** The densitometric analyses of figure 6D by Image J. Data are presented as the mean ± SD. Student's unpaired t-test. *, $P < 0.05$; **, $P < 0.01$; ***, $P < 0.001$.

Supplementary Figure S3. Overexpression of H2AFX could partially reverse the change of Vimentin mediated by miR-328-3p mimic.

Supplementary Figure S1.

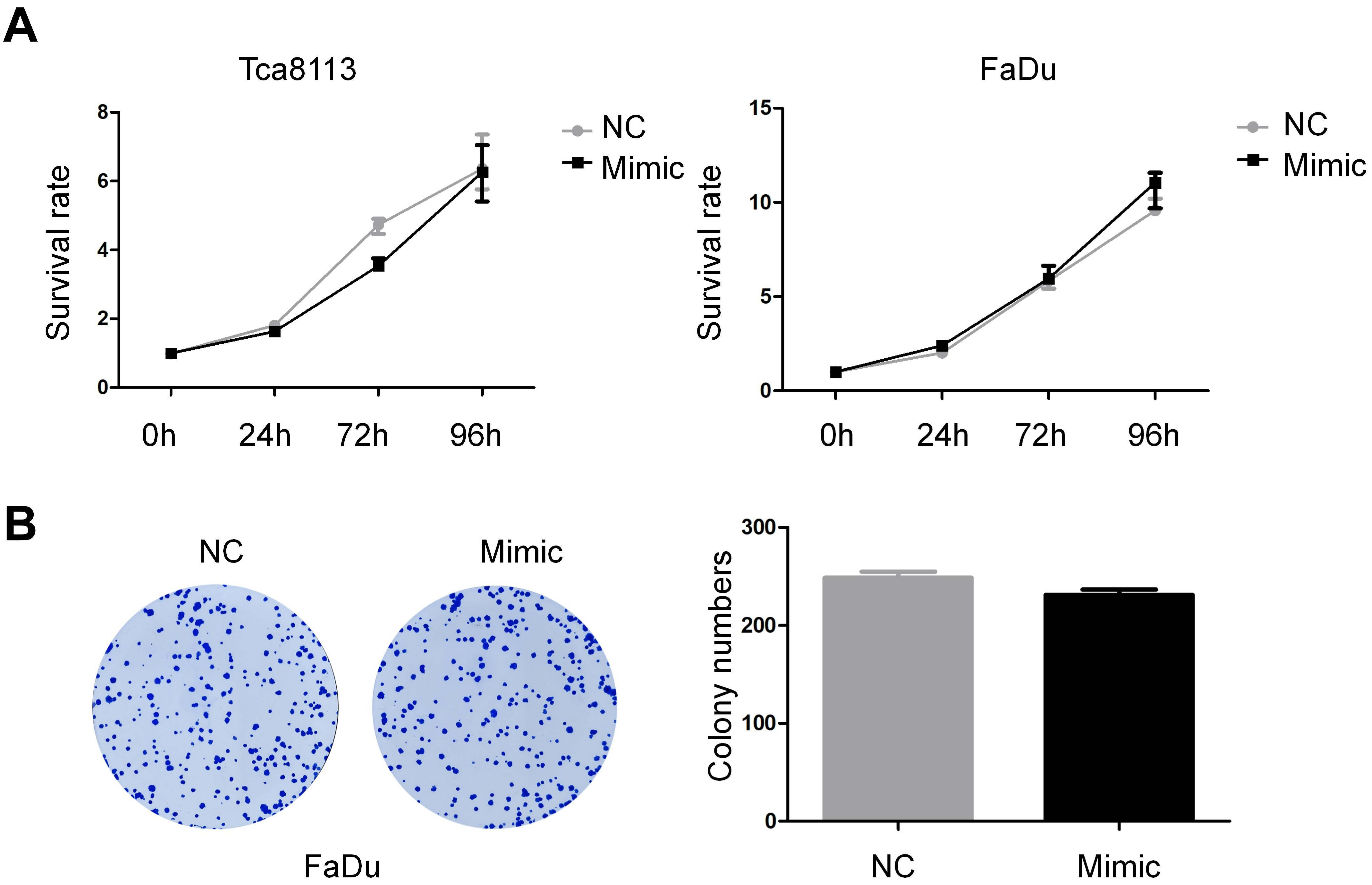

Supplementary Figure S2.

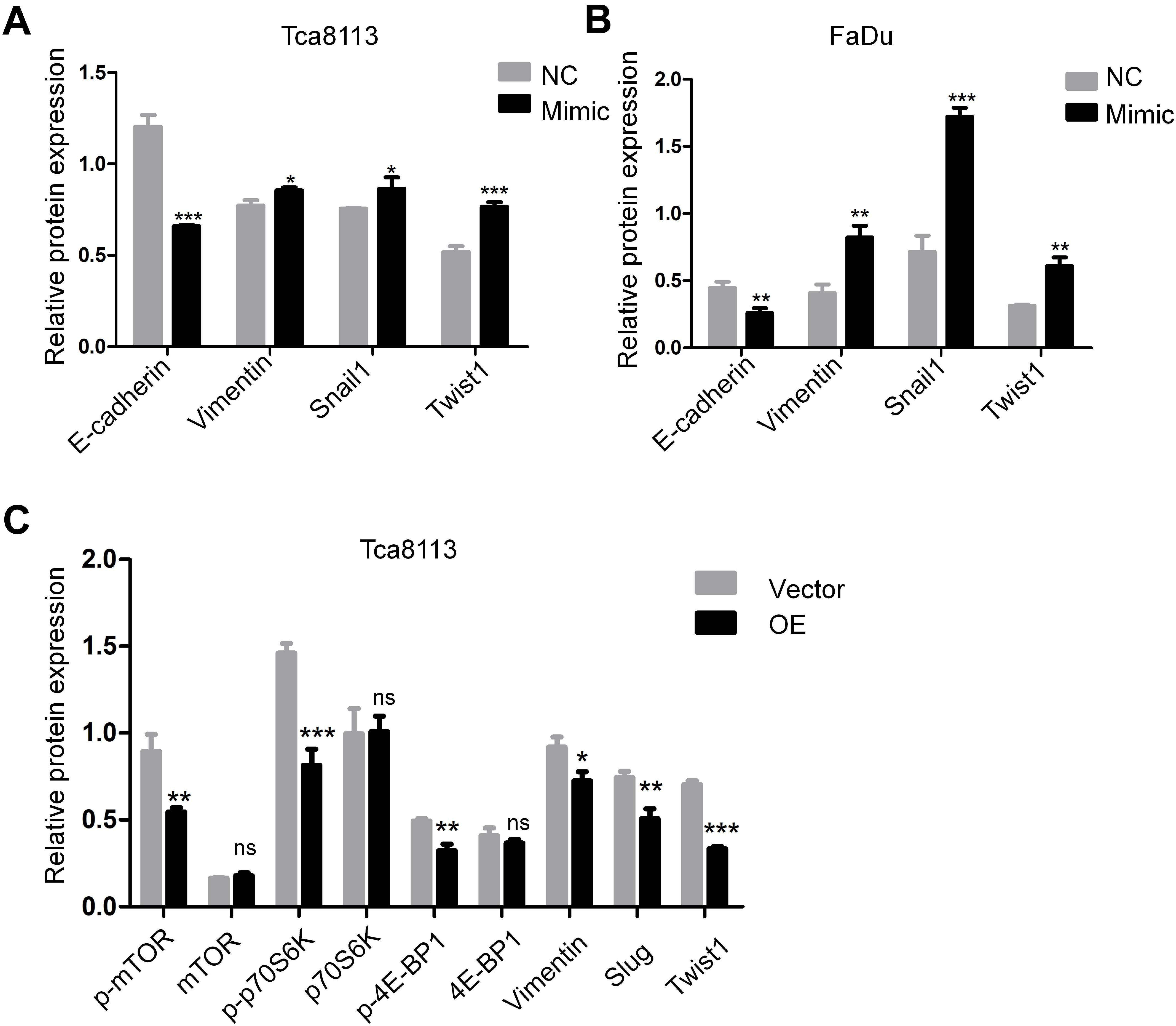

Supplementary Figure S3.

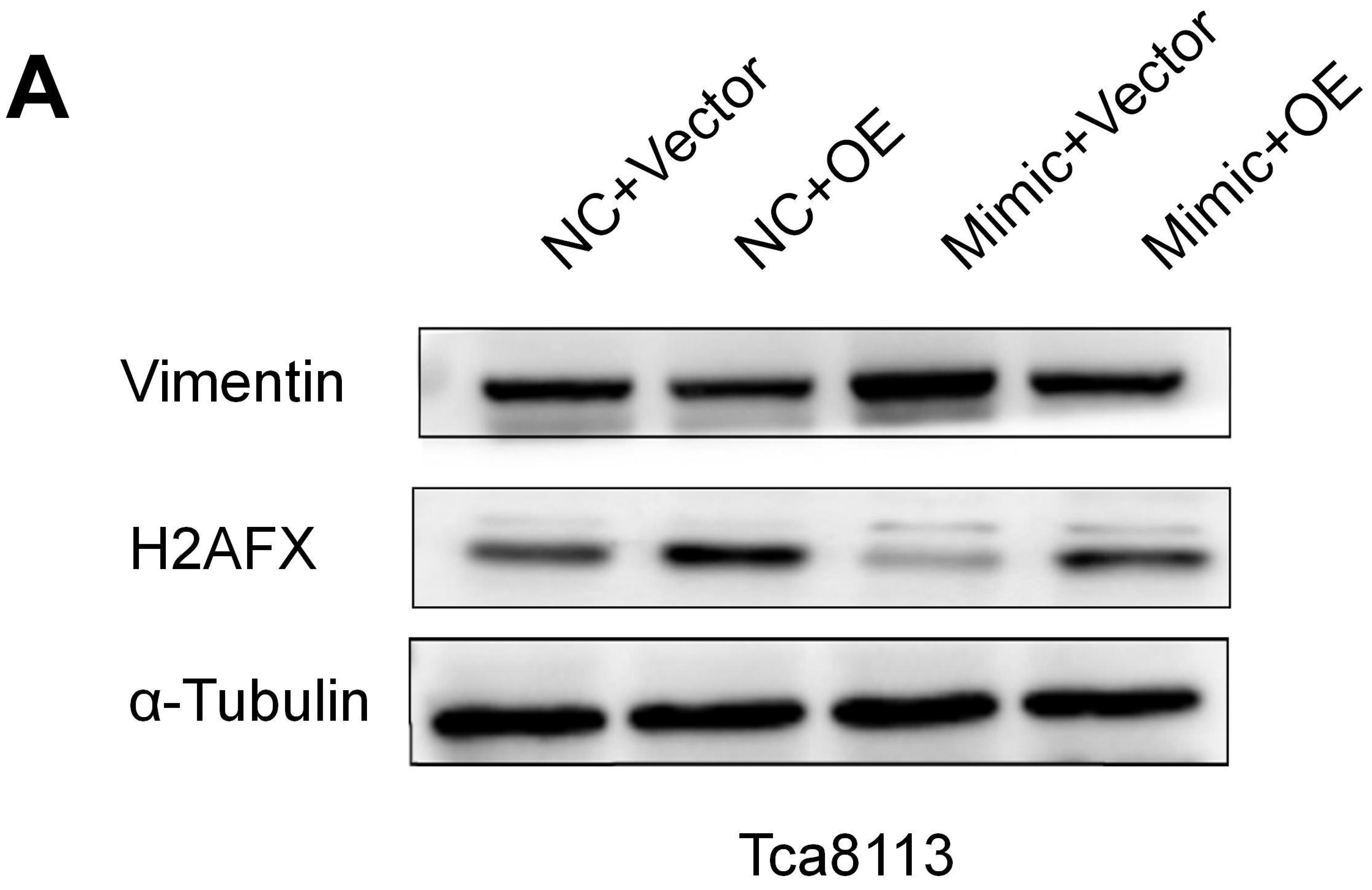

Table S1. The sequence of qRT-PCR primer

Gene name	Primer sequence (5’-3’)
GAPDH	
Forward	TCCAAAATCAAGTGGGGCGA
Reverse	AGTAGAGGCAGGGATGATGT
CDH1/E-cadherin	
Forward	TCCATTTCTTGGTCTACGCC
Reverse	CACCTTCAGCCAACCTGTTT
Vimentin	
Forward	GACAATGCGTCTCTGGCACGTCTT
Reverse	TCCTCCGCCTCCTGCAGGTTCTT
Twist1	
Forward	GCATTCTCAAGAGGTCGTGC
Reverse	ATGGTTTTGCAGGCCAGTTTG
Snail1	
Forward	CGAGTGGTTCTTCTGCGCTA
Reverse	GGGCTGCTGGAAGGTAAACT
Slug	
Forward	CTGTGACAAGGAATATGTGAGC
Reverse	CTAATGTGTCCTTGAAGCAACC
Fibronectin	
Forward	CCGTGGGCAACTCTGTC
Reverse	GGACTACCTGGAACAAAAGGG
N-cadherin	
Forward	CCGACGAATGGATGAAAGACC
Reverse	TTGCAGCCTATGCCAAAGC
Zeb1	
Forward	CTGCTCCCTGTGCAGTTACA
Reverse	GTGCACTTGAACTTGCGGTT
SIP1	
Forward	AGTCCATGCGAACTGCCATCTGAT
Reverse	CTGGACCATCTACAGAGGCTTGTA
mTOR	
Forward	GAGATACGCTGTCATCCCTTTA
Reverse	CTGTATTATTGACGGCATGCTC
PTEN	
Forward	GACCAGAGACAAAAAGGGAGTA
Reverse	ACAAACTGAGGATTGCAAGTTC
RPS6	
Forward	CCGCCAGTATGTTGTAAGAAAG
Reverse	TTCTCTTGGCCAAAAGTTTAGC
PRAS40	
Forward	GTCATCAGATGAGGAGAATGGG

Reverse	CTGGAAGTCGCTGGTGTTAAG
4E-BP1	
Forward	CAGGATCATCTATGACCGGAAA
Reverse	TCCATCTCAAACTGTGACTCTT
ITGA5	
Forward	CATGATGAGTTTGGCCGATTTG
Reverse	CCCCCAGGAAATACAAACACTA
FAM214B	
Forward	TGATGTTTCTGAGCAAAATGCC
Reverse	GAAGTCAAAGGTCACAAGGAAC
PIGA	
Forward	CGTCTTTTGGGAGCTTTAGAAC
Reverse	CCAACTCTGGTACTTACAACCT
LHFPL2	
Forward	CATGTGTGTACAGAGCATCATG
Reverse	AGAGAAGACAGCACAGATGAAA
CYP2S1	
Forward	GCTCCTGATGAAATACCCTCAT
Reverse	ATGAGGGTGCGGGGTATT
H2AFX	
Forward	ACGACGAGGAGCTCAACAAG
Reverse	TAGTACTCCTGGGAGGCCTG

Table S2. The antibody information

E-cadherin	Cat.3195
	1:1000 dilution, Cell Signaling Technology, Danvers, MA
Vimentin	Cat.5741
	1:1000 dilution, Cell Signaling Technology, Danvers, MA
TWIST1	Cat. 69366
	1:1000 dilution, Cell Signaling Technology, Danvers, MA
Slug	Cat. 9585
	1:1000 dilution, Cell Signaling Technology, Danvers, MA
Snail	Cat. 3879
	1:1000 dilution, Cell Signaling Technology, Danvers, MA
α-Tubulin	Cat. AC012
	1:5000 dilution, ABclonal Technology, Wuhan, China
GAPDH	Cat.AC002
	1:10000 dilution, ABclonal Technology, Wuhan, China
mTOR	Cat.2983
	1:1000 dilution, Cell Signaling Technology, Danvers, MA
Phospho-Mtor (Ser2448)	Cat.5536
	1:1000 dilution, Cell Signaling Technology, Danvers, MA
p70 S6 Kinase	Cat.BS3634
	1:500 ~ 1:1000 dilution, Bioworld Technology, USA
Phospho-p70 S6 Kinase (Thr389)	Cat.9234
	1:1000 dilution, Cell Signaling Technology, Danvers, MA
S6 Ribosomal Protein	Cat. 2217
	1:1000 dilution, Cell Signaling Technology, Danvers, MA
Phospho-S6 Ribosomal Protein (Ser235/236)	Cat.4858
	1:1000 dilution, Cell Signaling Technology, Danvers, MA
4E-BP1	Cat. 9644
	1:1000 dilution, Cell Signaling Technology, Danvers, MA
Phospho-4E-BP1	Cat. 2855
	1:1000 dilution, Cell Signaling Technology, Danvers, MA
Histone H2A.X	Cat. 7631
	1:1000 dilution, Cell Signaling Technology, Danvers, MA
Anti-rabbit IgG	Cat. 14708
	1:1000 dilution, Cell Signaling Technology, Danvers, MA
Anti-mouse IgG	Cat. 14709
	1:1000 dilution, Cell Signaling Technology, Danvers, MA
Anti-rabbit IgG (H+L), F(ab')2 Fragment	Cat. 4412
	1:500 dilution, Cell Signaling Technology, Danvers, MA
